# Time-Frequency-Based Separation of Earthquake and Noise Signals on Real Seismic Data: EMD, DWT and Ensemble Classifier Approaches

**DOI:** 10.3390/s25216671

**Published:** 2025-11-01

**Authors:** Yunus Emre Erdoğan, Ali Narin

**Affiliations:** Department of Electrical and Electronics Engineering, Faculty of Engineering, Zonguldak Bülent Ecevit University, Zonguldak 67100, Türkiye; y.emre.033@hotmail.com

**Keywords:** earthquake detection, time-frequency features, EMD, DWT, feature selection, machine learning

## Abstract

**Highlights:**

**What are the main findings?**

- Time-frequency features extracted using EMD, DWT, and combined EMD+DWT effectively separate earthquake and noise signals.
- Random Forest classifier with Lasso-selected EMD+DWT features achieved 100% accuracy, specificity, and sensitivity.

**What are the implication of the main findings?**

- Time-frequency-based feature extraction and selection improve real-time earth-quake detection.
- The approach provides a robust foundation for operational monitoring and ear-ly-warning systems.

**Abstract:**

Earthquakes are sudden and destructive natural events caused by tectonic movements in the Earth’s crust. Although they cannot be predicted with certainty, rapid and reliable detection is essential to reduce loss of life and property. This study aims to automatically distinguish earthquake and noise signals from real seismic data by analyzing time-frequency features. Signals were scaled using z-score normalization, and extracted with Empirical Mode Decomposition (EMD), Discrete Wavelet Transform (DWT), and combined EMD+DWT methods. Feature selection methods such as Lasso, ReliefF, and Student’s *t*-test were applied to identify the most discriminative features. Classification was performed with Ensemble Bagged Trees, Decision Trees, Random Forest, k-Nearest Neighbors (k-NN), and Support Vector Machines (SVM). The highest performance was achieved using the RF classifier with the Lasso-based EMD+DWT feature set, reaching 100% accuracy, specificity, and sensitivity. Overall, DWT and EMD+DWT features yielded higher performance than EMD alone. While k-NN and SVM were less effective, tree-based methods achieved superior results. Moreover, Lasso and ReliefF outperformed Student’s *t*-test. These findings show that time-frequency-based features are crucial for separating earthquake signals from noise and provide a basis for improving real-time detection. The study contributes to the academic literature and holds significant potential for integration into early warning and earthquake monitoring systems.

## 1. Introduction

Earthquakes are natural disasters caused by sudden energy releases within the Earth’s crust, leading to severe structural damage, economic losses, and loss of life. Their impact is particularly devastating in regions along active fault lines, leaving long-term societal and economic consequences. The detailed analysis of seismic waves is therefore essential for structural safety, urban planning, and disaster management strategies [1], while real-time seismic signal interpretation plays a critical role in both scientific research and post-disaster response.

Throughout history, major earthquakes have profoundly affected high-risk and densely populated areas, causing immense human and infrastructural losses. The 1999 Düzce earthquake in Türkiye (Mw 7.4) caused thousands of collapses and tens of thousands of deaths [2]; the 2004 Sumatra earthquake and tsunami (Mw 9.1) killed about 227,898 people and displaced millions [3]; the 2010 Haiti earthquake (Mw 7.0) resulted in over 230,000 deaths and 300,000 injuries [4]; and the 2011 Tōhoku earthquake and tsunami (Mw 9.0) in Japan caused 15,899 deaths and 6157 injuries [5]. Similarly, the 2015 Nepal (Mw 7.8) [6], 2018 Indonesia Sulawesi (Mw 7.5) [7], 2021 Haiti (Mw 7.2) [8], and 2023 Kahramanmaraş–Turkey-Syria (Mw 7.8) [9] earthquakes collectively demonstrated how magnitude, geology, and socio-economic factors jointly determine disaster impact, highlighting the importance of structural resilience, soil conditions, and effective disaster management.

In this context, real-time detection systems that activate immediately as earthquake waves begin to propagate are crucial. The instant identification of seismic signals through such systems can significantly reduce both loss of life and economic damage.

### 1.1. Literature Study

Artificial intelligence-supported systems process large amounts of data in a short time, providing fast and effective results in determining whether a signal is an earthquake or noise [10,11,12]. Such studies help understand earthquakes. In recent years, significant advances have been made in the literature with the intensive use of these technologies in earthquake-related studies [13,14,15].

Štajduhar et al. used a small subset of 150,000 three-component seismograms of the Local Earthquakes and Noise Database (LEN-DB) dataset and achieved 95.71% accuracy with AlexNet and Pseudo Wigner–Ville time-frequency representation [16]. Özkaya et al. used a subset of 10,002 data from LEN-DB and the most complicated lock pattern-based feature engineering model achieved 96.82% accuracy [17]. White et al. used a subset of Stanford Earthquake Dataset (STEAD) consisting of 65,536 samples, extracted features with FastMap and achieved 99% accuracy of signals with SVM classifier [18]. Cui et al. applied a Convolutional Neural Network (CNN) model with multi-scale attention mechanism and classified Texas Earthquake Dataset (TXED) earthquakes consisting of 20,000 samples with 99.83% accuracy [10]. Ertuncay et al. classified earthquake, vehicle and noise signals collected from Italy with a single CNN model and achieved 99.81% accuracy for earthquake detection [19]. In the study conducted with Indonesian Earthquake Seismic Monitoring (ESM) records, a total of 58 earthquake events from 3 stations were used. The earthquake signals were analyzed with different machine learning classifiers, namely SVM, k-NN, and DT, and the accuracy value achieved reached up to 92% [20]. Habbak et al. used 837 earthquake and quarry explosion events obtained from the Egyptian National Seismic Network; these data were processed with a multilayer CNN and attention mechanism to distinguish earthquake and explosion signals, and 100% accuracy was achieved despite the limited dataset [21]. Vasti and Dev. achieved 97% accuracy using Long Short Term Memory (LSTM) using 6000 3-component earthquake and noise signals from the STEAD [22]. In another study, Ertuncay et al. used 21,643 datasets to distinguish earthquakes from volcanic eruptions and achieved 99% accuracy using deep CNN [23]. These studies show that small data subsets combined with machine learning and deep learning techniques provide high accuracy and effective seismic event analysis [10,16,17,18,19,20,21,22,23].

### 1.2. Literature Gaps

Existing studies have achieved high classification accuracy on small subsets. However, most studies have focused on a limited number of examples. While some studies have evaluated three-component signals, single-component signals have generally been used. Their generalizability to larger datasets is questionable. Furthermore, significant shortcomings in the literature include the lack of combined use of time and frequency domain features in most studies, the lack of comprehensive comparison of feature selection methods, and the limited holistic performance analysis of different classifiers. This highlights the need to develop models that are comprehensive, generalizable, and suitable for real-time applications on larger-scale, multi-component datasets.

### 1.3. Motivation

While existing studies in the literature have achieved high accuracy on small subsets, their generalizability remains limited. Furthermore, they do not consider time- and frequency-domain features together, nor have they systematically compared feature selection methods and classifiers. The motivation for this study is to classify three-component earthquake and noise signals on a larger dataset of approximately 300,000 signals, evaluate time- and frequency-domain features together, and comprehensively compare different feature selection methods and classification algorithms. This aims to develop a model that is more generalizable, reliable, and applicable to practice compared to the small-scale, single-pronged approaches in the literature.

### 1.4. Innovations of the Study

**Figure 1:**
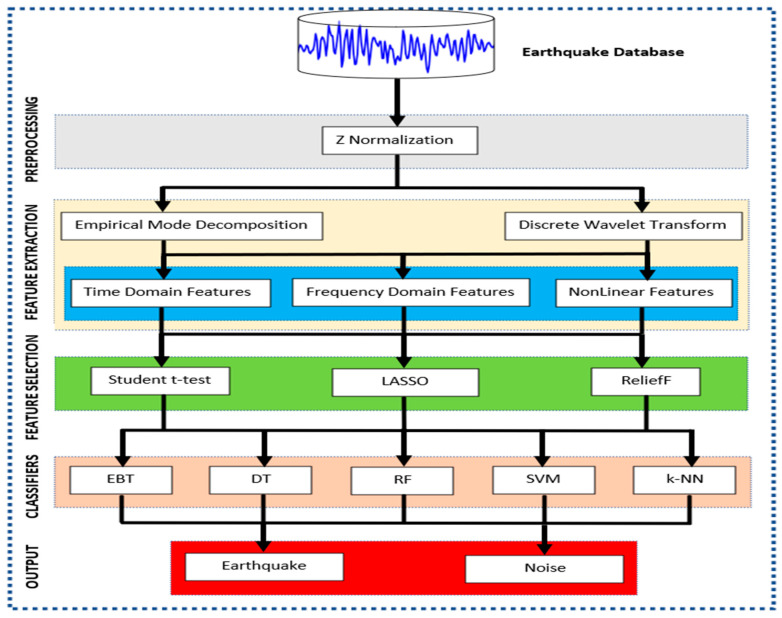
Flow Diagram of the Proposed System for Detection of Earthquake Signals.

The novelty of this study begins with the analysis of a large, three-component dataset; approximately 300,000 signal segments in the LEN-DB dataset were evaluated. While the literature mostly conducts one-dimensional or limited-scale analyses, this study considers both time and frequency domain features. Both amplitude and spectral features are taken into account with EMD, DWT, and EMD+DWT transformations. Lasso, ReliefF, and Student’s *t*-test methods were used in the feature selection phase, and 60 different classification scenarios were systematically evaluated with EBT, DT, RF, k-NN, and SVM algorithms. The results show that 100% Acc, 100% Spe, and 100% Rec are achieved when EMD+DWT features are selected with the Lasso method and classified with the RF algorithm. Furthermore, DWT and EMD+DWT features are found to offer higher accuracy compared to EMD alone. This approach demonstrates that the model can be integrated into real-time systems and has the potential for practical application thanks to its low computational cost and fast feature extraction. A detailed flowchart of the study is presented in Figure 1.

## 2. Materials and Methods

### 2.1. Dataset

LEN-DB, a comprehensive and high-quality open-access dataset containing seismic signals. The dataset is available at https://zenodo.org and is frequently referenced in scientific studies [24]. LEN-DB consists of three-component seismograms (east-X, north-Y, and vertical-Z) recorded by 1487 broadband or very broadband seismic stations located worldwide.

For each earthquake event, a time window of 27 s in total was created, starting 4 s before the theoretical arrival time of the P-wave. This window covers all seismic activity, including P, S, and surface waves. Signals were recorded with a sampling frequency of 20 Hz, and each seismogram contains 540 samples. The arrival times of the P-waves were calculated based on the IASP91 1-dimensional velocity model [25].

**Figure 2:**
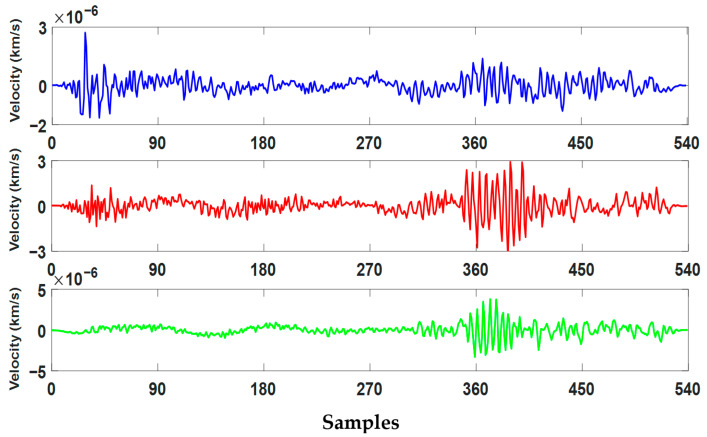
Three-component earthquake velocity signals recorded at station CN.MOBC, shown for X (blue), Y (red), and Z (green) directions. The event occurred on 21 May 2014 at 17:12:21 (UTC) with a magnitude of 2.8.

**Figure 3:**
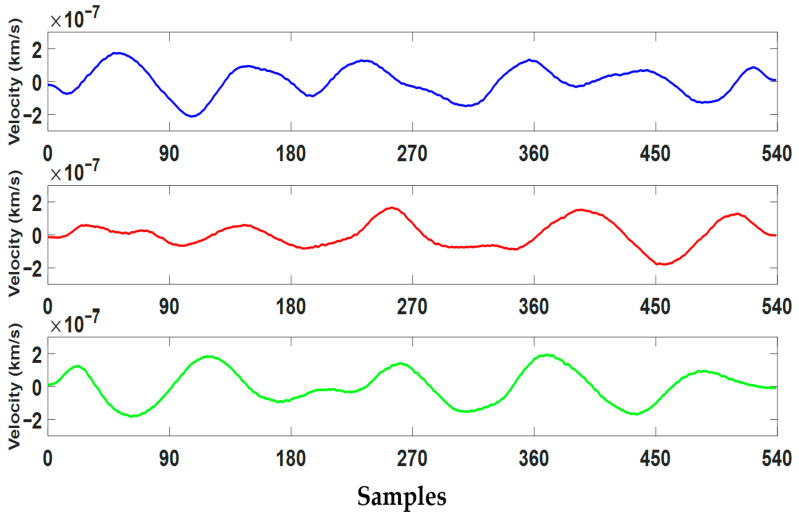
Three-component noise signals recorded at station OK.ELIS, shown for X (blue), Y (red), and Z (green) directions. The recording started on 22 March 2017 at 15:01:12 (UTC).

As a result of these processes, a total of 1,244,942 3-component seismograms were obtained for 304,878 earthquake events. 629,095 of these were labeled “EQ” (earthquake) and 615,847 were labeled “AN” (noise). There was no significant class imbalance in the dataset. In this study, a total of 300,000 3-component seismograms were analyzed using 150,000 randomly selected signals from each class. Sample earthquake signals are shown in Figure 2, while sample noise signals are shown in Figure 3. Summary statistics of the data structure are presented in Table 1.

### 2.2. Normalization

Normalization is the process of converting values in a dataset to a specific scale. The primary goal is to bring variables with different units of measurement or value ranges onto a common scale, resulting in more robust and comparable results in analysis and machine learning algorithms. Z-score normalization was used in this study. Z-score normalization is the process of rescaling each observation in a dataset according to its mean and standard deviation. This results in a mean of 0 and a standard deviation of 1. This method makes data at different scales comparable and improves the performance of machine learning algorithms. Its mathematical representation is as follows [26]:

(1) $$z=\frac{x-\mu }{\sigma }$$

Here, x represents any point in the dataset, μ represents the mean of the dataset, and σ represents the standard deviation of the dataset.

### 2.3. Feature Extraction

#### 2.3.1. Empirical Mode Decomposition

Empirical Mode Decomposition (EMD) is an adaptive and powerful method used for the analysis of nonlinear and non-stationary signals [27]. EMD decomposes complex signals into their natural oscillatory components, subcomponents called Intrinsic Mode Functions (IMFs). Each IMF represents different frequency bands of the signal and provides a detailed frequency-time resolution without destroying the original structure of the signal [28]. This feature distinguishes EMD from methods based on linear assumptions and makes it particularly suitable for the analysis of nonlinear signals such as natural, chaotic, and seismic.

An EMF decomposes a signal stepwise from high to low frequencies. Every EMF must satisfy two fundamental conditions: all local maxima and minima must be symmetrical about zero, and the signal’s average curve must be close to zero. These conditions ensure accurate extraction of subcomponents while preserving the signal’s inherent energy and oscillatory structure.

The steps followed when applying EMD on a signal x(t) are:

1. All extreme points of the signal are determined.
2. Upper and lower envelope curves are created by cubic spline interpolation of the extremum points.
3. The average of the envelope curves m(t) is calculated.
4. The difference signal d(t) = x(t) − m(t) is obtained.
5. The same procedure is repeated on the remaining signal m(t) and new IMFs are extracted.

**Figure 4:**
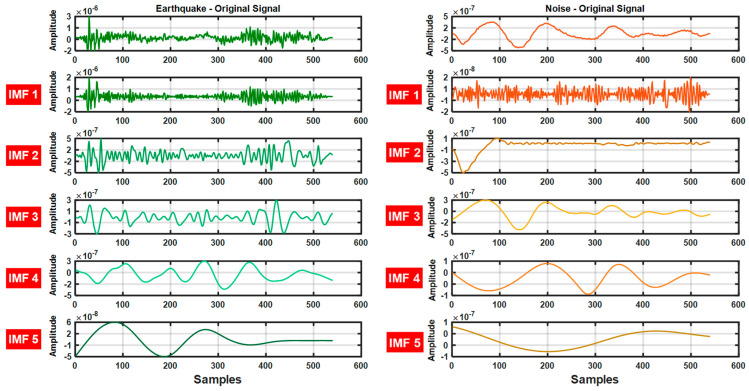
Earthquake and Noise data and 5-level IMF coefficients.

Time domain features (standard deviation, mean, variance, skewness), frequency domain features (LF, HF, LF/HF ratio, total power) and nonlinear features (Higuchi Fractal, Shannon Entropy, Renyi Entropy, Fuzzy Entropy, Tsallis Entropy, Teager Energy Operator (TEO) Mean) were calculated on the 5-level EMD signals obtained from the seismic data used in the study. Thanks to this comprehensive feature set, a total of 70 features were obtained for each signal and the earthquake signals were extracted from the noise. A rich dataset was created to provide high accuracy and performance in separation. The 5-level IMF signals obtained from the earthquake and noise data used in the study are shown in Figure 4.

#### 2.3.2. Discrete Wavelet Transform

Discrete Wavelet Transform (DWT) is a powerful method used to decompose time and frequency components of signals [29]. DWT significantly reduces computational overhead compared to Continuous Wavelet Transform (CWT) because CWT calculates coefficients at every combination of time and scale of the signal, while DWT only obtains coefficients at specific scales and time steps. This feature makes DWT particularly suitable for fast and efficient analysis of large datasets.

Wavelets can be thought of as short, time-limited oscillatory signals, allowing us to examine the local properties of the signal in both time and frequency. The most commonly used wavelet types in this context are Daubechies, Morlet, Haar, and Mexican Hat. DWT decomposes the signal into different frequency components and maps each component to the appropriate scale [30].

This allows for simultaneous analysis of high-frequency sudden changes and low-frequency, long-term components of the signal. One of the fundamental advantages of DWT is its ability to capture detailed time-frequency changes in the signal. This feature is particularly important in the analysis of nonlinear and non-stationary signals such as seismic data. The wavelet coefficients obtained from the DWT process represent the energy distribution and frequency characteristics of the signal at various scales. The wavelet function used in the DWT process is expressed as follows:

(2) $$\Psi_{m,n}\left(\frac{t-\tau }{s}\right)=s_{0}^{-\frac{m}{2}}\Psi \left(\frac{t-n\tau_{0}s_{0}}{s_{0}^{m}}\right)$$

Here, *m* and *n* are integer values representing the scale and time axis translation parameters. $s_{0}$ The fixed scale translation step $\tau_{0}$ is 2, and the time translation step is 1. These values are determined based on the most commonly used parameters in the literature and help optimize the performance of the DWT.

**Figure 5:**
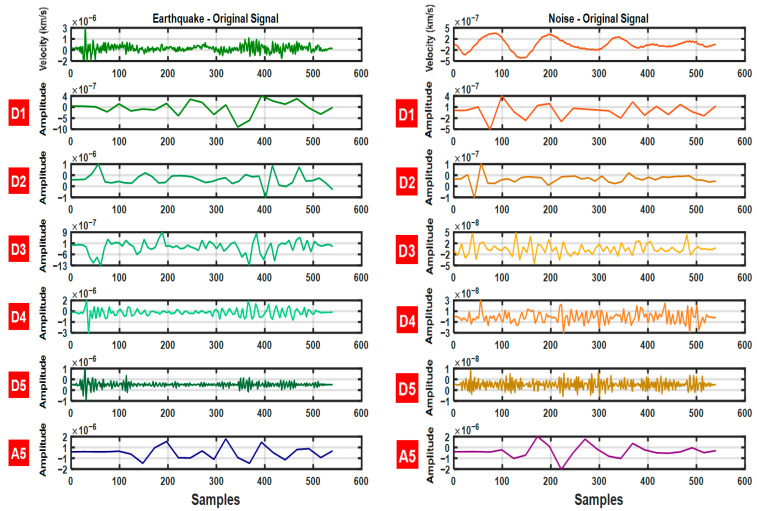
Earthquake and Noise data and 5-level DWT coefficients.

Similarly, for the 5-level DWT signals derived from the same seismic data, time-, frequency-, and nonlinear-domain features were computed, resulting in 84 features per signal. This rich feature representation facilitated the accurate discrimination of earthquake signals from environmental noise. The obtained DWT signals are illustrated in Figure 5.

### 2.4. Feature Selection

#### 2.4.1. ReliefF Algorithm

ReliefF is an example-based algorithm that provides efficient feature selection in high-dimensional and noisy datasets [31]. ReliefF, an improved version of the traditional Relief algorithm, is also compatible with multi-class datasets.

For each randomly selected sample, the algorithm compares the feature differences between the nearest neighbors of the same class (hit) and the nearest neighbors of different classes (miss) [32]. Feature weights are updated based on these differences. The goal is to highlight the features that best distinguish between classes.

The weight update for each feature A is calculated as follows:

(3) $$W\left[A\right]=W\left[A\right]-\frac{1}{m}\sum_{i=1}^{m}\;\left(diff\left(A,x_{i},{hit}_{i}\right)-\sum_{c\neq y_{i}}\;\;\frac{P\left(c\right)}{1-P\left(y_{i}\right)}\cdot diff\left(A,x_{i},{miss}_{i,c}\right)\right)$$

Here:

- *m*: number of randomly selected samples.
- $x_{i}$: *i*th example.
- $y_{i}$: *i*th example class.
- $P\left(c\right)$: probability of class c in the dataset.
- diff(*A*,*x,x*′): the difference in the feature *A* between *x* and *x′* (for continuous data, the absolute difference is usually taken).

The advantages of ReliefF include robustness to noise, compatibility with continuous and categorical features, consideration of interaction between features, and support for multi-class data. Its major drawback is that processing time can increase for large datasets. However, this can be mitigated by optimizing the number of samples and neighbors. ReliefF is widely used, particularly in fields such as seismic data analysis. The pseudocode for the ReliefF algorithm is shown in Algorithm 1.
**Algorithm 1** Pseudocode of the ReliefF algorithm**Input:** Feature matrix F with N instances and d=154 features extracted via EMD and DWT; class labels y∈{earthquake, noise}; parameters k (neighbors), m (sampled instances)**Output:** Feature importance scores $W\in R^{d}$**1:** Load the feature matrix F and class labels y
**2:** Initialize weights $W\left[A\right]=0.0$ for each feature A=1…d
**3:** For i=1 to m do**4:** Randomly select an instance $R_{i}$ from F
**5:** Find k nearest neighbors of the same class (hits) $H_{j}$, j=1…k
**6:** For each class $C\neq \mathrm{class}(R_{i})$ do**7:**  Find k nearest neighbors from class C (misses) $M_{j}(C)$, j=1…k
**8:** End for**9:** For each feature A=1…d do**10:**  $W[A]=W[A]-\frac{1}{m\cdot k}\sum_{j=1}^{k}diff(A,R_{i},H_{j})$**11:**  $W\left[A\right]=W\left[A\right]+\sum_{C\neq \mathrm{class}\left(R_{i}\right)}\frac{P\left(C\right)}{1-P\left(\mathrm{class}\left(R_{i}\right)\right)}\cdot \;\;\;\;\;\;\;\;\;\;\;\;\;\;\frac{1}{m\cdot k}\sum_{j=1}^{k}diff(A,{\;R}_{i},\;\;M_{j}(C))$**12:** End for**13:** End for**14:** Return the feature weights W as the importance scores

#### 2.4.2. Student *t*-Test

Student’s *t*-test is a classic hypothesis test used to determine whether the mean difference between two groups is statistically significant [33]. In the context of feature selection, it is applied to test how well each feature distinguishes two classes.

This test is particularly effective when the data are normally distributed and the features are independent of each other. The *t*-test statistic is calculated as follows:

t = (µ_1_ − µ_2_)/√((s_1_^2^/n_1_) + (s_2_^2^/n_2_)) (4)

Here, µ_1_ and µ_2_ are the class means, s_1_^2^ and s_2_^2^ are the class variances, and n_1_ and n_2_ are the sample sizes. The calculated t-value is compared to a specific significance level (e.g., 5%) to evaluate the distinctiveness of the feature.

The *t*-test’s greatest advantage is its simplicity and rapid computation. Therefore, it is widely preferred for initial feature elimination in large datasets [34]. Furthermore, it is relatively easy to interpret, as high t-values indicate strong differences between classes.

However, the test has some limitations. Most notably, it relies on the assumptions of normal distribution and independence. Failure to meet these conditions can lead to misleading results. Furthermore, it does not account for interactions between features, and multiple comparisons can increase the false-positive rate. Therefore, the results should be interpreted with caution.

With all these aspects, Student’s *t*-test is frequently used as a fast and practical method, especially in the first step of the feature pre-selection process. The pseudocode for Student’s *t*-test algorithm is shown in Algorithm 2.
**Algorithm 2** Pseudocode of the Student’s *t*-test algorithm**Input:** Feature matrix $F\in R^{N\times 154}$ extracted from signals using **EMD** and **DWT**; class labels y∈{Earthquake, Noise}; significance level α
**Output:** *t*-statistics t, *p*-values, and conclusion for each feature**1:** For each feature A=1…154, compute sample means:           ${\bar{X}}_{A}\;(\mathrm{Earthquake}),{\bar{Y}}_{A}\;(\mathrm{Noise})$
**2:** Compute sample variances:                $s_{X,A}^{2},s_{Y,A}^{2}$
**3:** Compute pooled standard deviation (for independent samples, equal variance):        $s_{p,A}=\sqrt{\frac{(n-1)s_{X,A}^{2}+(m-1)s_{Y,A}^{2}}{n+m-2}}$
**4:** Compute *t*-statistic for each feature:             $t_{A}=\frac{{\bar{X}}_{A}-{\bar{Y}}_{A}}{s_{p,A}\sqrt{\frac{1}{n}+\frac{1}{m}}}$
**5:** Compute degrees of freedom:              df=n+m−2
**6:** Determine *p*-value from *t*-distribution with df degrees of freedom**7:** Compare *p*-value to 0.05: If $p_{A}<0.05$, reject $H_{0}\Rightarrow$ feature A discriminates **Earthquake vs. Noise** Else, fail to reject $H_{0}\Rightarrow$ feature A not significant**8:** Return *t*-statistics, *p*-values, and significance conclusion for all 154 features

#### 2.4.3. LASSO (Least Absolute Shrinkage and Selection Operator)

LASSO is a powerful method that can simultaneously perform feature selection and model fitting [35]. It reduces the model coefficients by reducing some of them to zero, thus automatically eliminating unimportant features. In this way, it both prevents overfitting and simplifies the model.

The mathematical formula of LASSO is as follows:

(5) $${\hat{\beta }}^{\mathrm{lasso}\;}=\arg\underset{\beta }{min}\;\left\{\frac{1}{2n}\sum_{i=1}^{n}\;\;{\left(y_{i}-\beta_{0}-\sum_{j=1}^{p}\;\;x_{ij}\beta_{j}\right)}^{2}+\lambda \sum_{j=1}^{p}\;\;\left|\beta_{j}\right|\right\}$$

where

$y_{i}$: Dependent variable

$x_{ij}$: Independent variables

$\beta_{j}$: Coefficients

$\beta_{0}$: Constant term (intercept)

n: Number of samples

p: Number of variables

λ ≥ 0: The regularization parameter determines the intensity of the reduction in the coefficients.

Note: λ = 0 Lasso is equivalent to ordinary linear regression when, λ the coefficients become smaller as, and some become zero.

The key advantage of LASSO is its ability to perform feature selection and model learning in a single step. This feature allows it to operate efficiently on high-dimensional datasets and produce interpretable models. It also improves generalization performance by reducing the impact of noise and unnecessary variables.

However, it also has some limitations. It tends to select only one, especially when there are highly correlated features. This can lead to the exclusion of some important variables. Furthermore, because LASSO is a method based on linear relationships, it may be inadequate for data dominated by nonlinear structures.

As a result, LASSO is widely used as a simple, powerful, and effective feature selection method, especially for large and complex datasets [36]. It is suitable for high-dimensional classification problems such as seismic signal analysis. The pseudocode for the LASSO algorithm is shown in Algorithm 3.
**Algorithm 3** Pseudocode of the LASSO algorithm**Input:** Feature matrix $F\in R^{N\times 154}$(EMD + DWT features), class labels
y∈{Earthquake, Noise}, regularization parameter λ
**Output:** Selected feature coefficients β, feature importance1: Standardize the features in F to have zero mean and unit variance2: Initialize coefficients β=0 for all features3: Solve the LASSO optimization problem:          $\underset{\beta }{min}\frac{1}{2N}∥F\beta -y{∥}_{2}^{2}+\lambda ∥\beta {∥}_{1}$
4: Iterate until convergence:   Update $\beta_{j}$ for each feature using coordinate descent5: Identify selected features:   Features with non-zero $\beta_{j}$ are considered important6: Return β and feature importance ranking

### 2.5. Classification Algorithms

#### 2.5.1. Support Vector Machines

Support Vector Machine (SVM) is an effective and simple supervised learning method used in machine learning, especially in classification problems [37]. The main goal is to find the decision boundary (optimal hyperplane) that best separates the classes. This boundary is chosen to be equidistant from the closest examples (support vectors) to the classes, thus maximizing the margin. For linearly separable data, SVM directly finds this hyperplane with a wide margin; for nonlinearly separable data, linear separation is achieved by transforming the data to a higher-dimensional space using kernel functions. In this study, the regularization parameter C was scanned from 0.01 to 50 in 0.1 steps, and the kernel scale was modeled as 1 and the box constraint as 1. Kernel types were evaluated among linear, polynomial, and RBF options depending on the problem structure [38].

#### 2.5.2. K-Nearest Neighbor

The k-Nearest Neighbor (k-NN) algorithm is an intuitive and effective classification method frequently used in both statistical analysis and data mining [39]. This approach positions an unlabeled example in the feature space to determine its category and compares it with existing labeled data. This comparison is typically done by calculating the distances between data points; the Euclidean distance metric is most commonly used for this purpose. The algorithm evaluates the example to be classified based on the class labels of its k nearest neighbors in the training set. For example, when k = 1, the example is assigned only to the class of the nearest data point; when k = 5, the majority class among the five neighbors is assigned.

#### 2.5.3. Decision Trees

Decision trees (DTs) are hierarchical structures that classify data by branching [40]. The tree starts from the root node, where no decision has yet been applied. Each intermediate node applies a decision rule based on a specific feature in the dataset and divides the samples into sub-branches. This process continues until the data subsets become pure or another criterion is met. The nodes reached after the splitting is completed are called leaf nodes; each leaf usually contains the probability or class label for that class. In the classification process, the sample is directed to the appropriate branch according to the relevant feature at each decision point, starting from the root node, and finally, its class is determined by reaching a leaf [41].

#### 2.5.4. Random Forest

The RF algorithm is an ensemble learning method [42] that combines multiple decision trees. Different subsets are created from the training data using the bootstrap method, and a separate decision tree is trained on each. Furthermore, randomly selected feature subsets are used to determine the split point in each tree. These two randomization mechanisms prevent overfitting and increase generalization. Class prediction of the model is performed by summing the votes of all trees and selecting the class with the highest number of votes.

Random Forest provides higher accuracy and stability than a single decision tree. Combining independent decisions from different trees reduces errors. Furthermore, it is robust against overfitting in complex and high-dimensional datasets thanks to bootstrap sampling and random feature selection.

The algorithm can also work effectively with incomplete and noisy data. By measuring the importance of each feature, the model can determine which features contribute most to the classification. This provides advantages in model interpretation and feature engineering.

Disadvantages include increased computational and memory requirements when using multiple decision trees, and less transparency of the model’s structure compared to a single decision tree. However, current parallel processing technologies have greatly alleviated these problems.

#### 2.5.5. Ensemble Bagged Trees

DTs are supervised learning techniques widely used in classification and regression problems, offering a hierarchical structure to discover patterns in datasets [43]. DT models make decisions by branching based on specific feature conditions. In each branch, the data is split according to the features that provide the best separation. Entropy is used to evaluate this separation; entropy measures the uncertainty in the dataset, and low-entropy splits create more homogeneous groups.

The entropy formula is as follows:

(6) $$Entropy\left(S\right)=-\sum_{i=1}^{C}\;p_{i}\log_{2}\left(p_{i}\right)$$

where $p_{i},i$ represents S the probability of the ′th class, and represents the number of classes. C represents the set of all examples to be classified. Entropy is used to maximize information gain in branching the decision tree.

Information gain indicates how well an feature partitions the data and is calculated as follows:

(7) $$Information\;Gain\left(A\right)=Entropy\left(S\right)-\sum_{j=1}^{v}\;\frac{\left|S_{j}\right|}{\left|S\right|}Entropy\left(S_{j}\right)\;\;$$

where *v* is the number of subnodes formed according to feature *A*. $S_{j}$ is the number of samples in the *j*th subnode and Entropy ($S_{j}$) is the entropy of the *j*th subnode.

Ensemble learning is a method that improves classification performance by combining multiple learning algorithms. The EBT method combines multiple decision trees using the bagging technique [44].

### 2.6. Performance Criteria

A performance metric is a fundamental evaluation term that expresses how accurately the classification system used can classify data. One of the most important factors in these metrics is how the data is divided into training and test sets. The degree to which the training and test sets are separated from each other can directly affect the model’s performance. In the literature, “keep-apart,” “k-fold,” and “leave-one-out” cross-validation techniques are frequently preferred for dataset separation [45]. In this study, a 5-fold cross-validation (CV) strategy was employed to prevent potential overfitting and ensure the model’s generalization capability. The entire dataset was randomly divided into five non-overlapping subsets of approximately equal size. In each iteration, one subset was used for testing, while the remaining four were used for training, ensuring that no samples originating from the same event or station appeared simultaneously in both sets. This procedure was repeated five times, and the final performance metrics were obtained by averaging the results across all folds. This validation approach effectively minimizes bias and reduces the risk of overfitting. Additionally, during each iteration, True Positive (TP), True Negative (TN), False Positive (FP), and False Negative (FN) values were computed to assess model performance (Table 2).

As shown in Table 2, instances that are actually earthquake signals but are correctly identified by the algorithm as earthquake signals are designated as TP. Conversely, instances among real earthquake signals that the algorithm erroneously identifies as noise signals are designated as FN. Conversely, instances among real noise signals that the algorithm correctly categorizes as noise signals are designated as TN, while instances that are actually noise signals but are incorrectly evaluated as earthquake signals by the algorithm are designated as FP. In this study, performance metrics such as accuracy (Acc), sensitivity (Rec), specificity (Spe), precision (Pre), and F1-score (F1) are calculated.

(8) $$Acc=\frac{TP+TN}{TP+FN+FP+TN}$$

(9) $$Rec=\frac{TP}{TP+FN}$$

(10) $$Spe=\frac{TN}{TN+FP}$$

(11) $$Pre=\frac{TP}{TP+FP}$$

(12) $$F1=\frac{2*Pre*Rec}{Pre+Rec}$$

These performance metrics enable a comprehensive evaluation of the effectiveness of the classification models. Acc represents the overall correctness of the model, while Rec measures its ability to identify true positive instances. Spe quantifies the model’s capacity to correctly recognize negative cases, and Pre reflects the reliability of positive predictions. The F1-score serves as a harmonic balance between Rec and Pre, providing a more robust measure of performance in cases of class imbalance [46]. In addition, the Area Under the Curve (AUC) value derived from the ROC curves was also employed as a key performance indicator to further validate the model’s discriminative capability.

## 3. Results

In this study, all data processing and analysis steps were performed in the Matlab 2020 environment on a computer equipped with an Intel Core i5 processor and 12 GB of RAM. A large-scale dataset was used to separate earthquake and noise signals. This dataset, consisting of a total of 300,000 signals, includes a balanced mix of 150,000 earthquake signals and 150,000 noise signals. The primary reason for using this balanced dataset is to increase statistical reliability and establish a balance between computational cost and model performance. Given the dataset’s structure, which includes millions of samples, processing all the data would significantly increase hardware requirements and computational time. A representative sampling approach was adopted. The selected 150,000 samples provide sufficient diversity for both classes, providing a statistically significant representation of signal variations, minimizing model bias by preventing imbalance between classes, and aligning with data scales used in similar studies in the literature. This not only limits the computational burden encountered in extremely large datasets but also ensures reliable and comparable classification performance.

### 3.1. Performance Analysis of EMD and DWT Signals

In this section, the performances of different classifiers were examined using the data obtained directly with EMD, DWT and EMD+DWT without feature selection (Table 3).

In the EMD-based analysis, the highest performance was achieved with the RF algorithm. The RF classifier outperformed the other methods with an accuracy rate of 99.6330%, demonstrating superior performance. The EBT algorithm also demonstrated very successful results with an accuracy rate of 99.5907%. In contrast, the SVM and k-NN classifiers performed less well on the EMD data, reaching Acc values of 98.0567% and 98.2487%, respectively. These findings clearly demonstrate that tree-based methods are more advantageous in terms of accuracy and stability in the analysis of signals obtained with EMD (Figure 4).

**Figure 6:**
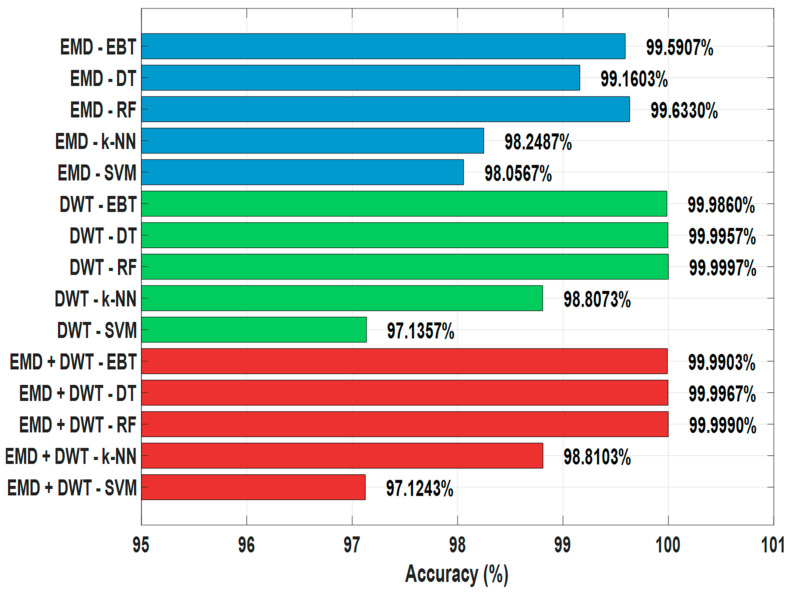
Comparisons of Feature Extraction Algorithms in Earthquake Detection.

In the DWT-based analysis, it is observed that all classifiers achieved very high performance. The RF, DT, and EBT classifiers achieved an accuracy rate of over 99.9860%. The RF algorithm, in particular, demonstrated remarkable performance with 99.9997% accuracy. In contrast, the SVM algorithm performed poorly with 97.1357% accuracy. The k-NN method demonstrated moderate performance with 98.8073% accuracy. This finding reveals that the DWT method provides a more robust representation in signal separation compared to EMD (Figure 6).

In the EMD+DWT combination, tree-based methods again stand out. DT, RF, and EBT algorithms achieved accuracy above 99.9903%. The RF classifier was also the most successful method in this section, with 99.9990% accuracy. k-NN and SVM performed less well, with 98.8103% accuracy and 97.1243% accuracy, respectively (Figure 6).

As a result, the analysis of the original signals shows that (Table 3, Figure 6). Tree-based algorithms (RF, DT, EBT) provided the highest performance for both EMD, DWT and EMD+DWT signals. DWT based signals have higher accuracy rates compared to EMD. SVM and k-NN classifiers showed lower success than other methods.

### 3.2. Performance Analysis of Feature Selection Algorithms

Three different feature selection techniques—Student’s *t*-test, ReliefF, and LASSO—were applied to both EMD- and DWT-based feature sets to identify the most discriminative attributes. Specifically, Student’s *t*-test selected 21 features from the EMD-based and 28 from the DWT-based features. The ReliefF algorithm retained 19 EMD-based and 25 DWT-based features, whereas the LASSO method selected 23 EMD-based and 30 DWT-based features for subsequent classification analysis. Each method used a different approach to determine the importance of features and eliminated unnecessary or low-information features. The LASSO method focused on reducing model complexity by specifically considering the correlation between features. The ReliefF algorithm evaluated the contribution of features to classification accuracy based on distances between samples. Student’s *t*-test was used for feature selection by testing the statistical significance of the mean differences between classes.

The results obtained by applying the LASSO feature selection method are shown in Table 4 and Figure 5. In EMD-based signals, the highest accuracy was achieved with the EBT (99.2510%) and RF (99.2370%) algorithms. DT, k-NN, and SVM achieved accuracies of 98.2637%, 98.4077%, and 97.8197%, respectively. These results reveal that tree-based methods are more advantageous than SVM and k-NN even after LASSO feature selection in EMD signals.

For DWT-based signals, the classifiers generally demonstrated very high performance. The RF algorithm achieved remarkable accuracy rates of 99.9993%, EBT 99.9907%, DT 99.9913%, k-NN 99.9753%, and SVM 99.9827%. This demonstrates that selecting DWT features with LASSO maximizes the success of the classifiers (Figure 5).

For the combined EMD+DWT signals, the LASSO-based feature selection method yielded the highest classification accuracy. Among the classifiers, the Random Forest (RF) algorithm achieved the best performance with 100% accuracy, followed by Ensemble Bagged Trees (EBT) with 99.9997%, Decision Tree (DT) with 99.9863%, k-Nearest Neighbors (k-NN) with 99.9587%, and Support Vector Machine (SVM) with 99.9507%. These results indicate that combining EMD and DWT features enables LASSO to extract the most informative and discriminative attributes, thereby reducing model complexity while enhancing classification performance. Moreover, this superior performance was achieved within a runtime of only 53 s, demonstrating that the proposed model attains excellent classification accuracy in a relatively short computational time and is well-suited for near real-time applications.

**Figure 7:**
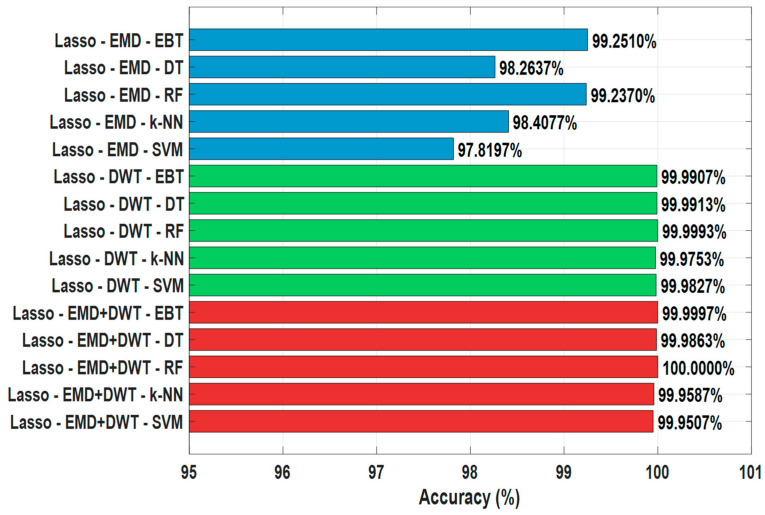
Comparison of LASSO Feature Selection Algorithm in Earthquake Detection.

Overall, LASSO feature selection significantly improves classification accuracy in earthquake data, particularly when used with tree-based algorithms such as RF and EBT, achieving nearly perfect results. DWT and EMD+DWT-based signals offer higher accuracy compared to EMD, while SVM and k-NN algorithms exhibit relatively lower performance than other methods. These results demonstrate that LASSO plays a critical role in the analysis of earthquake signals through feature selection and optimizes classification performance (Table 4, Figure 7).

**Figure 8:**
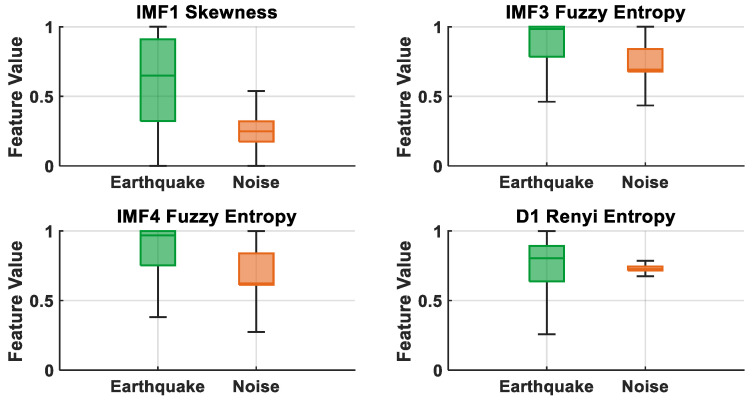
Boxplot representation of high discrimination features for LASSO.

Figure 8 presents box plots of the most discriminative features (IMF1 Skewness, IMF3 Fuzzy Entropy, IMF4 Fuzzy Entropy, and D1 Renyi Entropy) selected by the LASSO method. It is observed that the IMF1 Skewness feature has wider and higher values in earthquake signals, while it exhibits a lower and narrower distribution in noise signals. Although the IMF3 and IMF4 Fuzzy Entropy features show partial overlap between classes, earthquake signals generally have higher entropy values. D1 Renyi Entropy, on the other hand, exhibits a relatively lower distribution in earthquake signals and a higher and narrower distribution in noise signals, demonstrating a clear distinction. These results demonstrate that the features selected by the LASSO algorithm have a strong representational ability in distinguishing earthquake and noise signals.

The findings obtained from Student’s *t*-test-based feature selection are presented in Table 5 and Figure 8. This method allows the determination of the most effective features by evaluating the statistical significance of the mean differences between classes. In the analyses performed on EMD-based signals, the highest accuracy rates were achieved in the RF algorithm with 99.2937% and the EBT algorithm with 99.1287%, respectively. Among the other classifiers, DT showed accuracy values of 98.4570%, k-NN 97.8730%, and SVM 98.9203%. These findings reveal that, after feature selection performed with Student’s *t*-test method on EMD-based signals, tree-based algorithms, in particular, exhibited higher performance compared to other classifiers.

On DWT-based signals, RF demonstrated high performance with accuracy rates of 99.9990%, DT 99.9947%, EBT 99.9883%, k-NN 98.8063%, and SVM 97.1437%. These results demonstrate that selecting features obtained from DWT using the Student *t*-test method maximizes the success of the classifiers (Figure 8).

For combined EMD+DWT signals, the RF algorithm achieved the highest accuracy with 99.9987%, followed by EBT with 99.9900%, DT with 99.9897%, k-NN with 98.8140%, and SVM with 97.1150%. These findings demonstrate that using the combined EMD and DWT signals, Student’s *t*-test feature selection provides maximum information to the classifiers and significantly improves model performance (Table 5, Figure 8).

**Figure 9:**
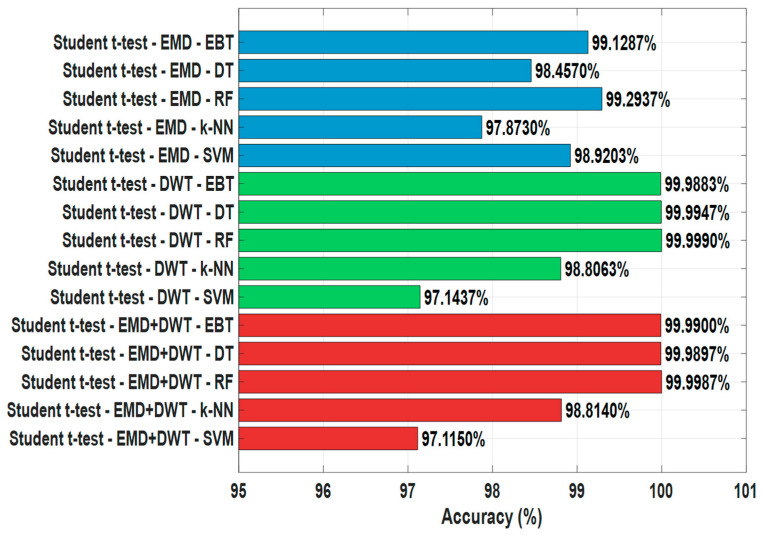
Comparison of Student’s *t*-test Feature Selection Algorithm in Earthquake Detection.

In general, Student’s *t*-test-based feature selection improves classification accuracy in earthquake data, providing significantly higher accuracy, especially when used in conjunction with tree-based algorithms such as RF and EBT. Furthermore, DWT and EMD+DWT-based signals offer higher accuracy compared to EMD alone, while the performance of SVM and k-NN algorithms is relatively lower than the other methods. These results demonstrate that Student’s *t*-test is an effective feature selection approach that optimizes performance in the classification of earthquake signals (Table 5, Figure 9).

**Figure 10:**
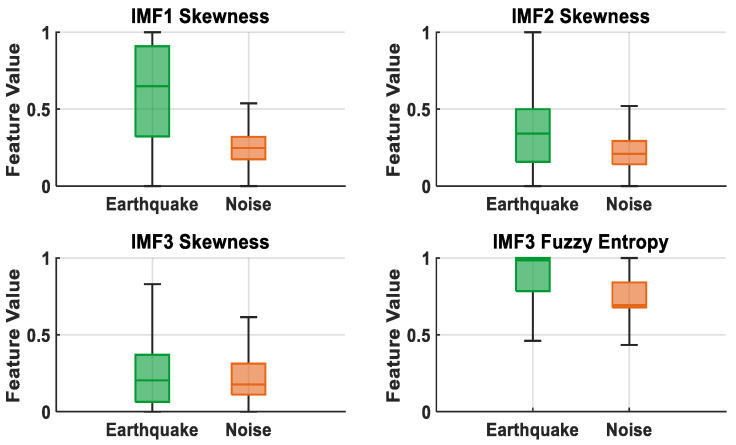
Box plot representation of features with high discrimination for Student’s *t*-test.

Figure 10 presents box plots of the most discriminative features (IMF1 Skewness, IMF2 Skewness, IMF3 Skewness, and IMF3 Fuzzy Entropy) selected using Student’s *t*-test method. It is observed that the IMF1 Skewness feature has wider and higher values in earthquake signals, while it exhibits a lower and narrower distribution in noise signals. While there is partial overlap between the IMF2 and IMF3 Skewness features, earthquake signals generally have higher entropy values. IMF3 Fuzzy Entropy, on the other hand, exhibits a relatively higher distribution in earthquake signals and a lower and narrower distribution in noise signals, demonstrating a clear distinction. These results demonstrate that the features selected by Student’s *t*-test algorithm have strong representativeness in distinguishing earthquake and noise signals.

**Figure 11:**
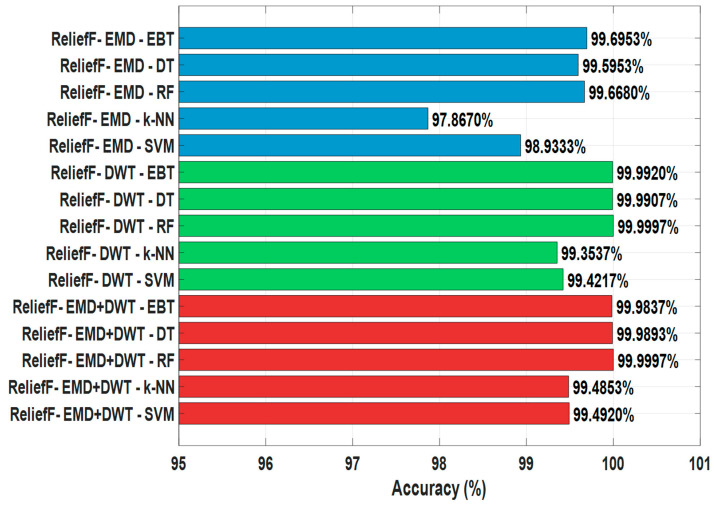
Comparison of ReliefF Feature Selection Algorithm with Earthquake Detection.

The findings obtained as a result of ReliefF feature selection are presented in Table 6 and Figure 11. This method enables the determination of the most effective features by evaluating the contribution of features to the classification success based on the distances between samples. For EMD-based signals, the highest accuracy values were achieved in EBT with 99.6953% and RF with 99.6680%. DT, with 99.5953%, k-NN with 97.8670%, and SVM with 98.9333% accuracy rates, exhibited lower performance compared to the highest-performing algorithms. These findings reveal that tree-based methods also exhibit superior performance compared to other classifiers after ReliefF feature selection in EMD-based signals.

For DWT-based signals, RF achieved high success with accuracy rates of 99.9997%, EBT 99.9920%, DT 99.9907%, k-NN 99.3537%, and SVM 99.4217%. These results show that selecting DWT features with the ReliefF method maximizes the performance of the classifiers (Figure 11).

For combined EMD+DWT signals, the RF algorithm achieved the highest accuracy with 99.9997%, followed by EBT with 99.9837%, DT with 99.9893%, k-NN with 99.4853%, and SVM with 99.4920%. These findings reveal that using the combined EMD and DWT signals, ReliefF feature selection provides more information to the classifiers, significantly improving the model’s performance (Table 6, Figure 11).

In general, ReliefF-based feature selection improves classification accuracy in earthquake data, providing significantly higher accuracy, especially when applied in conjunction with tree-based algorithms such as RF and EBT. Furthermore, DWT and EMD+DWT-based signals are observed to offer higher accuracy compared to EMD alone, while SVM and k-NN algorithms exhibit relatively lower performance than other methods. These results demonstrate that ReliefF is an effective feature selection approach that optimizes performance in the classification of earthquake signals (Table 6, Figure 11).

**Figure 12:**
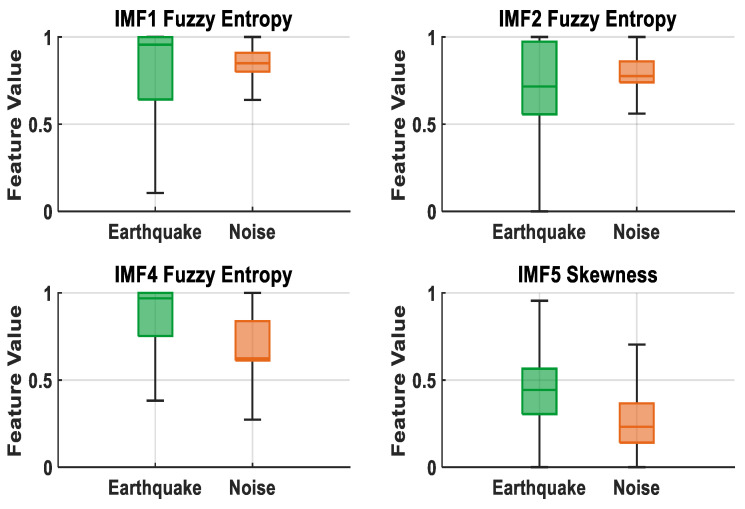
Boxplot representation of high discrimination features for ReliefF.

Figure 12 presents box plots of the most discriminative features (IMF1 Fuzzy Entropy, IMF2 Fuzzy Entropy, IMF4 Fuzzy Entropy, and IMF5 Skewness) selected by the ReliefF method. It is observed that the IMF1 Fuzzy Entropy feature has wider and higher values in earthquake signals, while it exhibits a lower and narrower distribution in noise signals. Although the IMF5 Skewness and IMF4 Fuzzy Entropy features show partial overlap between classes, earthquake signals generally have higher entropy values. IMF2 Fuzzy Entropy, on the other hand, exhibits a relatively low and broad distribution in earthquake signals and a higher and narrower distribution in noise signals, demonstrating a clear distinction. These results demonstrate that the features selected by the ReliefF algorithm have a strong representational ability in the separation of earthquake and noise signals.

**Figure 13:**
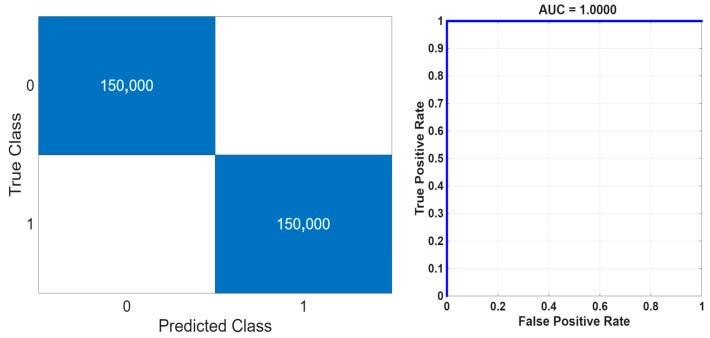
Confusion matrix and ROC curve of the RF algorithm using EMD+DWT features selected by the LASSO method.

A comparative analysis of the feature selection methods applied to earthquake data clearly demonstrates their influence on classification performance. For EMD-based signals, the highest accuracies were achieved using the ReliefF method in combination with the EBT (99.6953%) and RF (99.6680%) algorithms, whereas the LASSO and Student’s *t*-test methods yielded comparable but slightly lower results. For DWT-based signals, all methods exhibited high accuracy, with the ReliefF method coupled with the RF algorithm attaining the best performance (99.9997%). In the combined EMD+DWT feature set, the LASSO method with the RF algorithm achieved the maximum accuracy (100%), followed closely by the ReliefF method with the RF algorithm (99.9997%); both EBT and DT algorithms also demonstrated notably strong performance. Overall, the ReliefF method was particularly effective for EMD-based signals, while the LASSO method delivered superior results for DWT and EMD+DWT representations, offering the additional advantage of reducing model complexity. The confusion matrix and ROC curve corresponding to the highest-performing model are presented in Figure 13. Furthermore, Student’s *t*-test method effectively identified statistically significant features, thereby helping to maintain consistently high accuracy levels across all methods.

Beyond prior studies, these results demonstrate a distinct improvement in both accuracy and computational efficiency. Unlike previous works that relied primarily on deep learning architectures or limited feature domains, this study achieves near-perfect accuracy using a hybrid decomposition–selection–ensemble framework. This approach not only enhances model interpretability and generalization but also establishes a robust foundation for real-time earthquake detection systems.

## 4. Discussion

In recent years, the separation of seismic signals has become increasingly critical for improving the accuracy of earthquake early warning systems and ensuring structural safety. In this context, numerous machine learning and deep learning-based studies have been conducted to effectively distinguish earthquake signals from ambient noise. Table 7 provides a comprehensive summary of selected works in the literature, detailing their datasets, applied methodologies, and achieved classification accuracies. This comparison offers a clear perspective on the performance of existing approaches and highlights potential areas for further improvement.

When compared with recent studies in the literature, the proposed EMD+DWT-based framework demonstrates superior performance in earthquake signal classification. Štajduhar [16] achieved 95.71% accuracy using AlexNet with Pseudo Wigner–Ville distributions, while Özkaya [17] and White [18] reported accuracies of 96.82% and 99.0% using MCLP and FastMap+SVM models, respectively. More recently, Cui [10] and Ertuncay [19] obtained high accuracies (99.83% and 99.81%) by employing deep learning architectures with multi-scale and convolutional layers, whereas Vasti and Dev [22] achieved 97.0% using an LSTM model. Although these approaches show promising results, they often rely on computationally demanding deep networks and large-scale training data.

In contrast, this study achieved 100% accuracy using a hybrid EMD+DWT feature extraction approach combined with LASSO feature selection and a Random Forest classifier, applied to an extensive dataset of 300,000 seismograms from LEN-DB. The integration of EMD and DWT provided complementary time–frequency representations, while LASSO effectively reduced dimensionality by retaining only the most informative features. As a result, the proposed model not only surpassed the performance of deep learning-based methods [10,19,20,21,22,23] but also achieved this outcome with significantly lower computational cost and shorter runtime.

Compared with the literature, the main contributions of this study are:

1. Achieving high accuracy on a larger, three-component dataset.
2. Evaluating time and frequency domain features together.
3. Feature selection and systematic comparison of different classifier combinations.
4. Ensemble methods provide high generalization capability and stable performance.

## 5. Conclusions

In this study, earthquake and noise signals were accurately separated using hybrid EMD+DWT features, LASSO-based selection, and an RF classifier. Evaluation on a large dataset of 300,000 signals achieved 100% accuracy, specificity, and recall demonstrating the robustness and high discriminative power of the proposed framework. The integration of feature decomposition, selection, and ensemble learning also enhanced computational efficiency, highlighting its potential for real-time earthquake early warning systems.

Despite these achievements, the dataset is limited to a specific region and seismic condition, and performance under diverse environmental and sensor conditions remains to be validated. Real-time deployment also requires further optimization of computational cost and latency.

Future work will explore metaheuristic feature selection, multi-region and multi-sensor datasets, and hardware-level implementations to enable real-time earthquake detection. These efforts aim to establish a reliable, interpretable, and scalable foundation for next-generation seismic early warning systems.

## Figures and Tables

**Table 1:** Data used in the study and their characteristics.

Class	Number of Data	Total	Data Length (Samples)
Earthquake	150,000	300,000	540
Noise	150,000		540

**Table 2:** Confusion Matrix.

		Predicted Class	
		Negative	Positive
**Real Class**	**Negative**	TN True Negative	FP False Positive
	**Positive**	FN False Negative	TP True Positive

**Table 3:** Comparisons of Feature Extraction Algorithms for Earthquake Detection.

Feature Extraction Method/s	Classifier	Runtime (s)	Acc (%)	Spe (%)	Pre (%)	Rec (%)	F1 (%)
EMD	EBT	72	99.5907	99.3580	99.3610	99.8233	99.5916
EMD	DT	15	99.1603	99.1620	99.1620	99.1587	99.1603
EMD	RF	65	99.6330	99.4100	99.4126	99.8560	99.6338
EMD	k-NN	28	98.2487	98.9613	98.9463	97.5360	98.2361
EMD	SVM	136	98.0567	97.3687	97.4044	98.7447	98.0699
DWT	EBT	83	99.9860	99.9767	99.9767	99.9953	99.9860
DWT	DT	18	99.9957	99.9960	99.9960	99.9953	99.9957
DWT	RF	75	99.9997	99.9993	99.9993	100.0000	99.9997
DWT	k-NN	33	98.8073	98.1467	98.1708	99.4680	98.8152
DWT	SVM	171	97.1357	95.6720	95.7951	98.5993	97.1770
EMD+DWT	EBT	160	99.9903	99.9900	99.9900	99.9907	99.9903
EMD+DWT	DT	36	99.9967	99.9967	99.9967	99.9967	99.9967
EMD+DWT	RF	151	99.9990	99.9980	99.9980	100.0000	99.9990
EMD+DWT	k-NN	55	98.8103	98.1493	98.1735	99.4713	98.8181
EMD+DWT	SVM	321	97.1243	95.6853	95.8060	98.5633	97.1651

**Table 4:** Comparison of LASSO Feature Selection Algorithm in Earthquake Detection.

Feature Extraction Method/s	Classifier	Runtime (s)	Acc (%)	Spe (%)	Pre (%)	Rec (%)	F1 (%)
EMD	EBT	24	99.2510	98.9807	98.9861	99.5213	99.2530
EMD	DT	6	98.2637	98.2927	98.2917	98.2347	98.2632
EMD	RF	22	99.2370	98.9667	98.9722	99.5073	99.2391
EMD	k-NN	28	98.4077	98.9827	98.9708	97.8327	98.3985
EMD	SVM	49	97.8197	96.6887	96.7619	98.9507	97.8441
DWT	EBT	26	99.9907	99.9873	99.9873	99.9940	99.9907
DWT	DT	6	99.9913	99.9900	99.9900	99.9927	99.9913
DWT	RF	26	99.9993	99.9993	99.9993	99.9993	99.9993
DWT	k-NN	12	99.9753	99.9613	99.9613	99.9893	99.9753
DWT	SVM	59	99.9827	99.9747	99.9747	99.9907	99.9827
EMD+DWT	EBT	56	99.9997	100.0000	100.0000	99.9993	99.9997
EMD+DWT	DT	13	99.9863	99.9867	99.9867	99.9860	99.9863
**EMD+DWT**	**RF**	**53**	**100.0000**	**100.0000**	**100.0000**	**100.0000**	**100.0000**
EMD+DWT	k-NN	17	99.9587	99.9253	99.9254	99.9920	99.9587
EMD+DWT	SVM	116	99.9507	99.9673	99.9673	99.9340	99.9507

**Table 5:** Comparison of Student’s *t*-test Feature Selection Algorithm in Earthquake Detection.

Feature Extraction Method/s	Classifier	Runtime (s)	Acc (%)	Spe (%)	Pre (%)	Rec (%)	F1 (%)
EMD	EBT	22	99.1287	99.3100	99.3075	98.9473	99.1271
EMD	DT	5	98.4570	98.4107	98.4121	98.5033	98.4577
EMD	RF	21	99.2937	99.4860	99.4840	99.1013	99.2923
EMD	k-NN	26	97.8730	99.0507	99.0278	96,6953	97.8477
EMD	SVM	46	98.9203	98.6173	98.6257	99.2233	98.9236
DWT	EBT	25	99.9883	99.9947	99.9947	99.9820	99.9883
DWT	DT	5	99.9947	99.9927	99.9927	99.9967	99.9947
DWT	RF	24	99.9990	99.9993	99.9993	99.9987	99.9990
DWT	k-NN	11	98.8063	98.1173	98.1429	99.4953	98.8145
DWT	SVM	59	97.1437	95.7087	95.8284	98.5787	97.1841
EMD+DWT	EBT	53	99.9900	99.9967	99.9967	99.9833	99.9900
EMD+DWT	DT	11	99.9897	99.9907	99.9907	99.9887	99.9897
EMD+DWT	RF	50	99.9987	100.0000	100.0000	99.9973	99.9987
EMD+DWT	k-NN	15	98.8140	98.1467	98.1711	99.4813	98.8219
EMD+DWT	SVM	109	97.1150	95.6433	95.7679	98.5867	97.1568

**Table 6:** Comparison of ReliefF Feature Selection Algorithm with Earthquake Detection.

Feature Extraction Method/s	Classifier	Runtime (s)	Acc (%)	Spe (%)	Pre (%)	Rec (%)	F1 (%)
EMD	EBT	21	99.6953	99.9067	99.9063	99.4840	99.6947
EMD	DT	4	99.5953	99.6087	99.6086	99.5820	99.5953
EMD	RF	20	99.6680	99.9300	99.9296	99.4060	99.6671
EMD	k-NN	24	97.8670	99.0493	99.0263	96.6847	97.8415
EMD	SVM	43	98.9333	99.2233	99.2188	98.6433	98.9302
DWT	EBT	24	99.9920	99.9887	99.9887	99.9953	99.9920
DWT	DT	5	99.9907	99.9920	99.9920	99.9893	99.9907
DWT	RF	23	99.9997	99.9993	99.9993	100.0000	99.9997
DWT	k-NN	10	99.3537	99.2447	99.2463	99.4627	99.3544
DWT	SVM	57	99.4217	99.2367	99.2395	99.6067	99.4227
EMD+DWT	EBT	51	99.9837	99.9807	99.9807	99.9867	99.9837
EMD+DWT	DT	10	99.9893	99.9893	99.9893	99.9893	99.9893
EMD+DWT	RF	48	99.9997	99.9993	99.9993	100.0000	99.9997
EMD+DWT	k-NN	14	99.4853	99.3260	99.3281	99.6447	99.4862
EMD+DWT	SVM	105	99.4920	99.2973	99.3001	99.6867	99.4930

**Table 7:** Earthquake-Noise Classification Studies in the Literature.

Study	Dataset	Number of Data	Model	Acc (%)
Štajduhar (2022) [16]	LEN-DB	150,000 seismograms	AlexNet (Pseudo Wigner–Ville)	95.71
Özkaya (2023) [17]	LEN-DB	10,002 seismograms	MCLP	96.82
White (2023) [18]	STEAD	65,536 seismograms	FastMap+SVM	99.00
Cui (2025) [10]	TXED	20,000 seismograms	CNN + Multi-scale Attention	99.83
Ertuncay (2025) [19]	Italy	Earthquake, vehicle, noise	CNN	99.81
Indonesian ESM (2006–2009) [20]	3 stations	58 earthquake events	SVM, k-NN, DT	92.00
Habbak (2024) [21]	Egyptian National Seismic Network	837 earthquake and quarry explosion	CNN	100
Vasti & Dev (2025) [22]	STEAD	6000 earthquakes and noise	LSTM	97.00
Ertuncay (2024) [23]	Italy	21,643 earthquakes and volcanic eruptions	DCNN	99.00
**This study**	**LEN-DB**	**300,000 seismograms**	**EMD+DWT/Lasso/RF**	**100**

## Data Availability

We used publicly available dataset called the “LEN-DB”. The dataset is available at https://zenodo.org [24].
